# S-nitrosylation of the scaffold protein STRAP enhances oxidative stress–induced apoptosis

**DOI:** 10.1016/j.jbc.2026.111141

**Published:** 2026-01-08

**Authors:** Weixiong Xu, Daniel Chen, Hua-Lin Zhou

**Affiliations:** Department of Biochemistry and Molecular Biology, Medical College of Georgia, Augusta University, Augusta, Georgia

**Keywords:** S-nitrosylation, nitric oxide, inducible nitric oxide synthase, serine–threonine kinase receptor–associated protein, apoptosis signal–regulating kinase 1, apoptosis

## Abstract

Serine–threonine kinase receptor–associated protein (STRAP) functions as a negative regulator of apoptosis by inhibiting apoptosis signal–regulating kinase 1 (ASK1) activity. STRAP is consistently present in the inducible nitric oxide synthase (iNOS) interactome and contains two essential cysteine residues, Cys152 and Cys270, which are required for its interaction with ASK1. However, the role of the STRAP–iNOS interaction remains unclear. In this study, we found that STRAP specifically interacts with iNOS but not with endothelial NOS or neuronal NOS. iNOS mediates the S-nitrosylation of STRAP, which disrupts the STRAP–ASK1 interaction, increases ASK1 activity, activates the mitogen-activated protein kinase kinase 3 (MKK3) and mitogen-activated protein kinase (p38) pathway, and enhances hydrogen peroxide–induced apoptosis. Notably, Cys152 and Cys270 are also the primary S-nitrosylation sites of STRAP. Mutation of these residues to serine (STRAP-C152/270S) abolishes the STRAP–ASK1 interaction, constitutively activates the ASK1-MKK3–p38 pathway, and increases apoptosis. Moreover, iNOS overexpression fails to promote hydrogen peroxide–induced apoptosis in STRAP-C152/270S–expressing cells, underscoring the essential role of STRAP S-nitrosylation in iNOS–mediated cell death. This study provides the first evidence that S-nitrosylation of STRAP is critical for the regulation of apoptosis and uncovers a novel cell survival mechanism mediated by the iNOS-SNO–STRAP-ASK1 signaling axis.

Nitric oxide (NO) is synthesized from l-arginine by a family of nitric oxide synthases (NOSs) in mammals, including neuronal NOS (nNOS), inducible NOS (iNOS), and endothelial NOS (eNOS) (1). NO plays a dual role in apoptosis, acting either as an inhibitor or promoter depending on the cellular context and NO concentration (2, 3). At low physiological concentrations, NO produced by eNOS and nNOS inhibits apoptosis. In contrast, high and pathological levels of NO generated by iNOS promote apoptosis (4). The activity of eNOS and nNOS is tightly regulated by phosphorylation, calcium, and calmodulin, ensuring that NO production remains within physiological and homeostatic limits (5). However, iNOS is not subject to such regulation and can produce large amounts of NO when induced by inflammatory cytokines, bacterial infections, or other immune responses (6). This unregulated NO production leads to pathological nitrosative stress and apoptosis.

The activity of NO is largely mediated through S-nitrosylation, a redox reaction in which NO reacts with thiol groups to form nitrosothiols (SNO) (7). S-nitrosylation is a signaling post-translational modification that regulates protein activity, interactions, stability, and subcellular localization (8). While many studies have elucidated the molecular mechanisms by which NO causes apoptosis—including the intrinsic and extrinsic pathway activation (9, 10, 11), p53 accumulation (12), and DNA damage (13), it remains unclear whether iNOS-mediated protein S-nitrosylation plays a direct role in regulating apoptosis.

Serine–threonine kinase receptor–associated protein (STRAP) is a scaffolding protein that exerts its functions through interactions with a wide range of binding partners (14). One of the main roles of STRAP is to reduce cellular sensitivity to apoptosis, thereby promoting cell survival. Mechanistically, STRAP interacts with apoptosis signal–regulating kinase 1 (ASK1) in a redox-dependent manner and inhibits its activity. ASK1, a member of the mitogen-activated protein kinase kinase kinase family, is a key regulator of apoptosis, and its inactivation can directly suppress apoptotic signaling (15, 16). ASK1 is activated by various stress stimuli, such as tumor necrosis factor α, Fas signaling, hydrogen peroxide (H_2_O_2_), and DNA damage (17). Once activated, ASK1 phosphorylates and activates mitogen-activated protein kinase kinases (MKKs), which in turn activate c-Jun N-terminal kinase (JNK) and p38 MAPK (18). The ASK1–MKK–JNK/p38 signaling cascade amplifies stress signals and induces apoptosis (19). NO has also been shown to activate ASK1 and induce apoptosis (9); however, the mechanism by which NO activates the ASK1 pathway remains unclear.

Under normal physiological conditions, STRAP binds to ASK1 and inhibits its activity, thereby deactivating the ASK1–MKK-p38 pathway and suppressing apoptosis (15). However, this interaction is disrupted by external stimuli, including H_2_O_2_, tumor necrosis factor-α, and endoplasmic reticulum stress, leading to increased ASK1 activity and promotion of cell death (15). Two cysteine residues, Cys152 and Cys270, within STRAP are required for its interaction with ASK1. Mutation of these cysteines disrupts the interaction with ASK1 and increases cell apoptosis, indicating that Cys152 and Cys270 play a critical role in the function of STRAP (15). However, whether these residues can be modified by S-nitrosylation and contribute to iNOS-mediated apoptosis remains unknown.

In this study, we found that STRAP interacts specifically with iNOS but not with eNOS or nNOS. iNOS mediates S-nitrosylation of STRAP at Cys152 and Cys270, which disrupts its interaction with ASK1 and enhances H_2_O_2_-induced apoptosis. These findings highlight the critical role of STRAP S-nitrosylation in apoptosis regulation and uncover a novel mechanism by which iNOS-derived NO promotes cell death.

## Results

**Figure 1:**
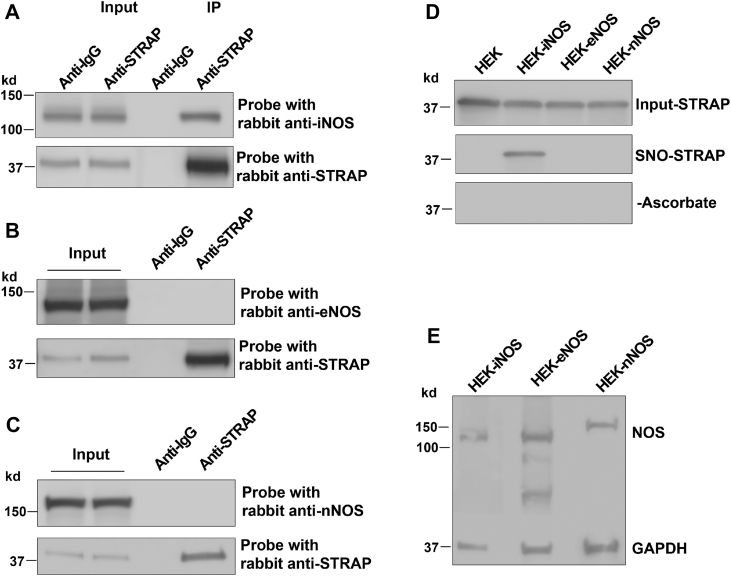
**Interaction between STRAP and three NOS memebers.***A*–*C*, coimmunoprecipitation analysis of STRAP interaction with iNOS (*A*), eNOS (*B*), or nNOS (*C*). HEK293 cells were cotransfected with FLAG-STRAP and iNOS, eNOS, or nNOS expression plasmids. Cell lysates were immunoprecipitated with anti-FLAG antibody or control mouse immunoglobulin G, followed by immunoblotting with anti-iNOS, anti-eNOS, or anti-nNOS antibodies. n = 2. *D*, detection of S-nitrosylated STRAP (SNO-STRAP) in HEK293 cells overexpressing iNOS, eNOS, or nNOS. Reaction without ascorbate (−Ascorbate) was included as a negative control. n = 3. *E*, expression levels of iNOS, eNOS, and nNOS in HEK293 cells. eNOS, endothelial nitric oxide synthase; HEK293, human embryonic kidney 293 cell line; iNOS, inducible nitric oxide synthase; NOS, nitric oxide synthase; nNOS, neuronal nitric oxide synthase; SNO, S-nitrosolthiol; STRAP, serine–threonine kinase receptor–associated protein.

STRAP has been identified in the iNOS interactome databases (20, 21), prompting us to investigate whether STRAP can be S-nitrosylated by iNOS and to explore the functional consequences of this interaction. To validate the interaction and determine whether STRAP also associates with other NOS family members, we coexpressed iNOS, eNOS, or nNOS with STRAP in human embryonic kidney (HEK) cells. Coimmunoprecipitation (IP) assays revealed that STRAP specifically interacts with iNOS but not with eNOS or nNOS (Fig. 1, *A*–*C*).

We next examined whether STRAP undergoes S-nitrosylation. In WT HEK cells, as well as in eNOS- or nNOS-overexpressing HEK cells, no SNO signal was detected for STRAP. However, a strong SNO signal for STRAP was observed in iNOS-overexpressing HEK cells, indicating that STRAP is specifically S-nitrosylated by iNOS (Fig. 1, *D* and *E*).

**Figure 2:**
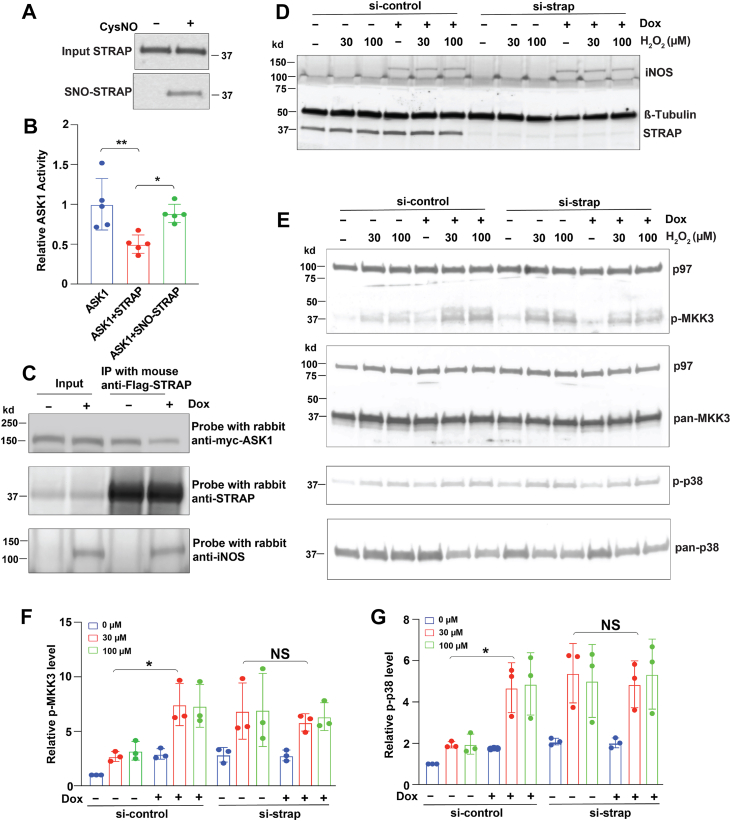
**S-nitrosylation of STRAP abolishes its ability to suppress ASK1 activity and disrupts their interaction.***A*, S-nitrosylated STRAP (SNO-STRAP) after *in vitro* SNO donor Cys-NO treatment. *B*, ASK1 kinase activity in the presence of unmodified STRAP or SNO-STRAP. n = 5. *C*, interaction between STRAP and ASK1 following iNOS induction. FLAG-STRAP and Myc-ASK1 were coexpressed in HEK-iNOS cells. Cell lysates were immunoprecipitated with anti-FLAG antibody and probed with anti-Myc antibody. “−” indicates the absence of doxycycline (Dox); “+” indicates the presence of Dox. n = 3. *D*, expression of iNOS and STRAP in HEK-iNOS cells transfected with no-targeting negative control siRNA or STRAP siRNA and induced by Dox. β-tubulin was used as a loading control. *E*, phosphorylation levels of MKK3 (Ser189) and p38 (Thr180/Tyr182) in HEK-iNOS cells transfected with control siRNA or STRAP siRNA. Cells were treated with H_2_O_2_, with or without Dox induction (“−” or “+”). n = 3. *F* and *G*, quantification of MKK3 phosphorylation and p38 phosphorylation levels in *E*. Phosphorylation levels of MKK3 and p38 (ImageJ signal intensities in *E*) were first normalized to the corresponding pan-MKK3 and pan-p38 levels, respectively, and then were normalized to the si-control condition without Dox (−) and H_2_O_2_ treatment (0 μM). All data in *B*, *F*, and *G* are presented as mean ± SD. Each data point represents one biological replicate. Statistical significance was determined using one-way ANOVA followed by Tukey’s *post hoc* test. ∗*p* < 0.05 and ∗∗*p* < 0.01. ASK1, apoptosis signal–regulating kinase 1; H_2_O_2_, hydrogen peroxide; HEK, human embryonic kidney cell line; iNOS, inducible nitric oxide synthase; MKK, mitogen-activated protein kinase kinase; NO, nitric oxide; SNO, S-nitrosolthiol; STRAP, serine–threonine kinase receptor–associated protein.

We then investigated the functional impact of STRAP S-nitrosylation. Given that STRAP is known to bind and inhibit ASK1 activity (15), we tested whether S-nitrosylation affects this regulatory function. STRAP was first S-nitrosylated *in vitro* using a high concentration of the SNO donor CysNO to generate SNO-STRAP (Fig. 2*A*). ASK1 activity was then assessed using an *in vitro* assay. In the presence of unmodified STRAP, ASK1 activity was reduced by approximately 50% (Fig. 2*B*), consistent with its known inhibitory role. However, SNO-STRAP failed to inhibit ASK1, showing ASK1 activity levels comparable to those observed in the absence of STRAP (Fig. 2*B*). These results suggest that S-nitrosylation abolishes STRAP's ability to suppress ASK1 activity.

The interaction between STRAP and ASK1 is known to be critical for the inhibition of ASK1 activity (15). To determine whether iNOS affects this interaction, STRAP and ASK1 were coexpressed in HEK cells with doxycycline (Dox)-inducible iNOS expression. We found that iNOS expression reduced the interaction between STRAP and ASK1 (Fig. 2*C*), suggesting that iNOS-mediated STRAP S-nitrosylation disrupts its binding to ASK1. This disruption likely explains why SNO-STRAP loses its ability to inhibit ASK1 activity.

To assess whether iNOS-mediated STRAP S-nitrosylation also regulates ASK1 activity at the cellular level, we examined the phosphorylation levels of MKK3 and p38, two downstream substrates of ASK1. In iNOS-overexpressing HEK cells transfected with no-targeting negative control siRNA, iNOS expression induced by Dox increased phosphorylation of MKK3 and p38 after oxidative stress treatment (H_2_O_2_; 30 μM and 100 μM) (Fig. 2, *D*–*G*), confirming that S-nitrosylation of STRAP by iNOS results in a loss of its inhibitory function on ASK1 in cells.

To validate that iNOS-mediated ASK1 activation depends on STRAP, we knocked down STRAP using siRNA. In STRAP-deficient cells without iNOS expression, phosphorylation of MKK3 and p38 was already elevated after oxidative stress treatment (H_2_O_2_; 30 μM and 100 μM), consistent with STRAP's inhibitory effect on ASK1 activity. Importantly, iNOS expression in STRAP-knockdown cells had no additional effect on MKK3 or p38 phosphorylation (Fig. 2, *D*–*G*), demonstrating that STRAP is required for iNOS-mediated ASK1 activation.

**Figure 3:**
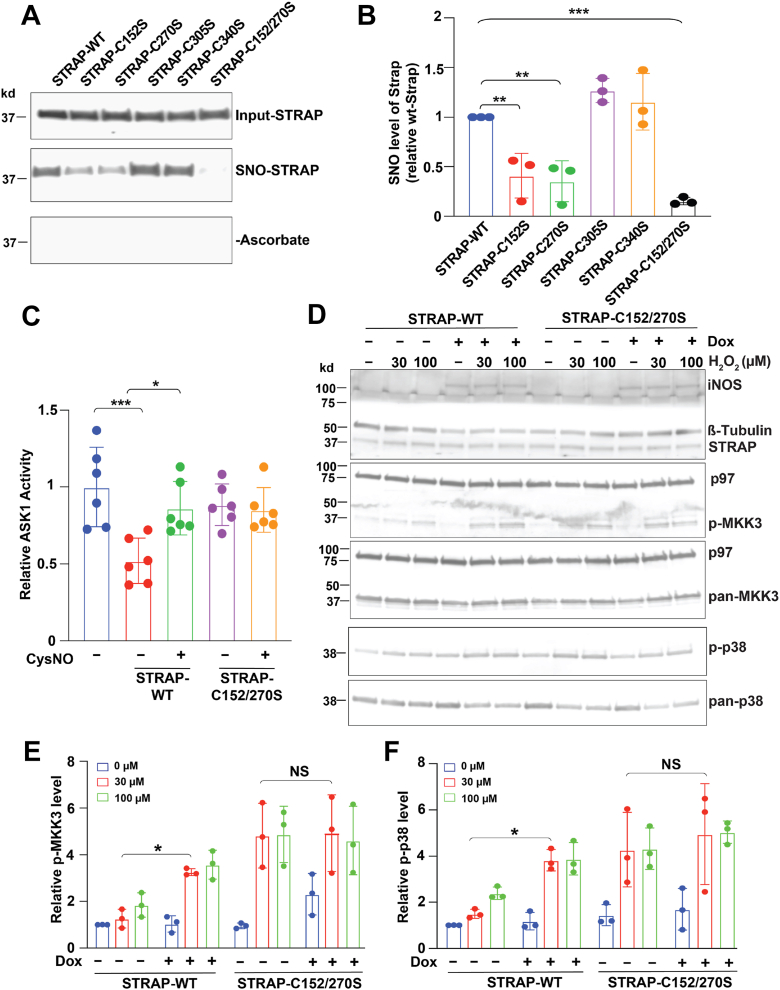
**Mutation of SNO sites in STRAP alters ASK1 activity.***A*, detection of S-nitrosylated STRAP (SNO-STRAP) in HEK-iNOS cells overexpressing WT STRAP or candidate SNO-site mutants (C152S, C270S, C305S, C340S, and C152/270S). Cells were induced with doxycycline (Dox). Reaction without ascorbate (−Ascorbate) was used as a negative control. n = 3. *B*, quantification of SNO-STRAP levels shown in *A*. SNO level of all samples (ImageJ signal intensities in *A*) was first normalized to the corresponding input, respectively, and then were normalized to the SNO level of STRAP-WT. *C*, ASK1 kinase activity in the presence of STRAP-WT or STRAP-C152/270S, with or without CysNO treatment (“−” or “+”). ASK1 activity in all samples was normalized to the average ASK1 activity of the first column (without STRAP protein). *D*, phosphorylation of MKK3 (Ser189) and p38 (Thr180/Tyr182) in HEK-iNOS cells expressing STRAP-WT or STRAP-C152/270S, following H_2_O_2_ treatment with or without Dox induction (“−” or “+”). The p97 was used as a loading control. n = 3. *E* and *F*, quantification of MKK3 phosphorylation and p38 phosphorylation levels in *D*. Phosphorylation levels of MKK3 and p38 (ImageJ signal intensities in *D*) were first normalized to the corresponding pan-MKK3 and pan-p38 levels, respectively, and then normalized to the STRAP-WT condition without Dox (−) and H_2_O_2_ treatment (0 μM). All data in *B*, *E*, and *F* are presented as mean ± SD, with each data point representing one biological replicate. Statistical significance was determined using one-way ANOVA followed by Tukey’s *post hoc* test. ∗*p* < 0.05, ∗∗*p* < 0.01, and ∗∗∗*p* < 0.001. ASK1, apoptosis signal–regulating kinase 1; H_2_O_2_, hydrogen peroxide; HEK, human embryonic kidney cell line; iNOS, inducible nitric oxide synthase; MKK3, mitogen-activated protein kinase kinase; SNO, S-nitrosolthiol; STRAP, serine–threonine kinase receptor–associated protein.

To dissect the role of S-nitrosylated STRAP (SNO-STRAP) in regulating ASK1 activity, we first identified the S-nitrosylated cysteine residues (SNO sites) in STRAP. STRAP contains four conserved cysteines: Cys152, Cys270, Cys305, and Cys340. Each cysteine was individually mutated to serine to assess its contribution to SNO level. In HEK293 cells, mutation of either Cys152 or Cys270 (STRAP-C152S and STRAP-C270S) reduced SNO level by approximately 60% (Fig. 3, *A* and *B*). The double mutation (STRAP-C152/270S) almost completely abolished the SNO signal (Fig. 3, *A* and *B*), indicating that Cys152 and Cys270 are the primary SNO sites in STRAP.

Next, we investigated whether mutation of the SNO sites affects ASK1 activity *in vitro*. Purified STRAP-WT and STRAP-C152/270S were treated with the NO donor CysNO. Unmodified STRAP-WT reduced ASK1 activity by approximately 50%, whereas CysNO-treated STRAP-WT failed to inhibit ASK1 activity. In contrast, both unmodified and CysNO-treated STRAP-C152/270S had no effect on ASK1 activity (Fig. 3*C*), confirming that S-nitrosylation of STRAP at Cys152 and Cys270 is essential for its inhibitory effect on ASK1.

To examine the effect of SNO site mutation (C152/270S) in STRAP on the ASK1–MKK–p38 pathway, cells expressing either STRAP-WT or the SNO-deficient mutant STRAP (STRAP-C152/270S) were treated with H_2_O_2_ (30 μM and 100 μM). In STRAP-WT cells, iNOS expression led to increased phosphorylation of both MKK3 and p38 (Fig. 3, *D*–*F*), consistent with previous findings (Fig. 2*E*). In contrast, STRAP-C152/270S mutant cells showed elevated basal phosphorylation of MKK3 and p38, likely because of the mutant’s inability to interact with and inhibit ASK1 (Fig. 3, *D*–*F*). Notably, iNOS expression in STRAP-C152/270S cells did not further enhance phosphorylation, indicating that S-nitrosylation of STRAP at C152/270 is a critical regulatory step in modulating the ASK1–MKK–p38 signaling pathway.

**Figure 4:**
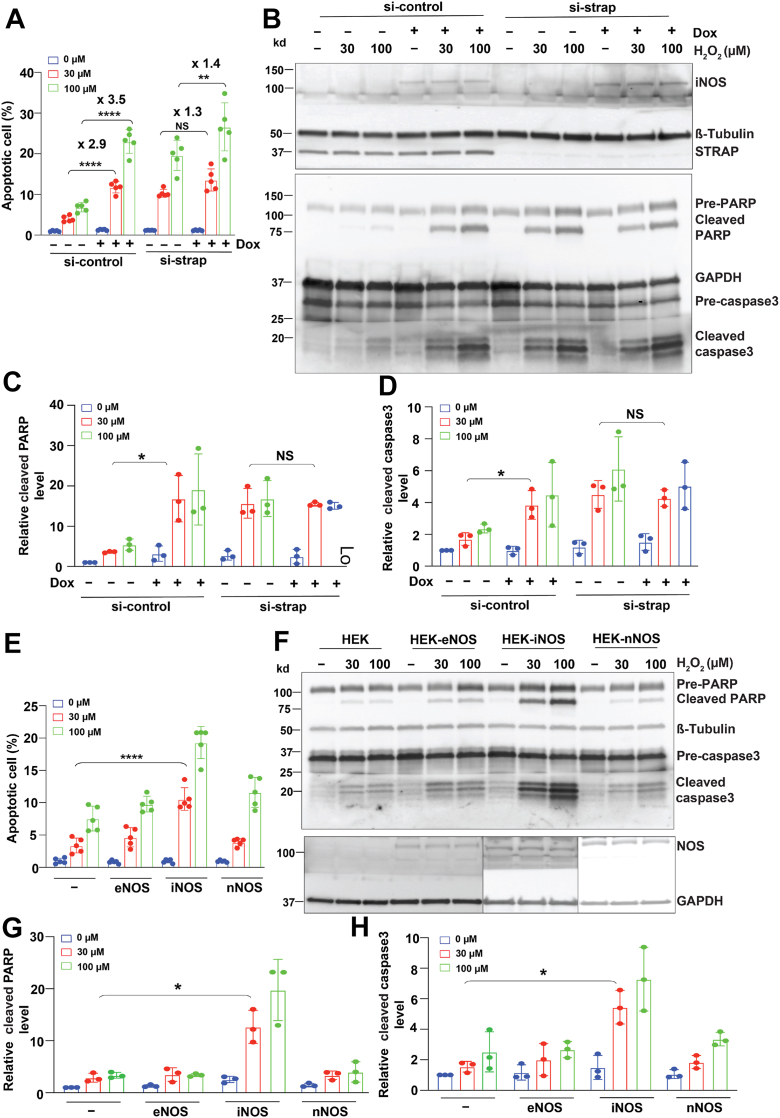
**iNOS-mediated STRAP S-nitrosylation enhances sensitivity to H_2_O_2_-induced apoptosis.***A*, apoptosis in si-control and si-STRAP knockdown HEK-iNOS cells was measured using the RealTime-Glo Annexin V Apoptosis Assay. Cells were transfected with control or STRAP-targeting siRNA, induced with doxycycline (Dox) (“+”), and treated with H_2_O_2_. All samples were normalized to the average apoptotic cell percentage of si-control without Dox induction (−) and H_2_O_2_ treatment (0 μM). *B*, cleavage of PARP and caspase 3 in si-control or si-STRAP knockdown HEK-iNOS cells following Dox induction (“+”) and H_2_O_2_ treatment. Expression levels of iNOS and STRAP are shown in the *upper panel*. β-tubulin and GAPDH were used as a loading control. n = 3. *C* and *D*, quantification of cleaved PARP and cleaved caspase 3 levels in *B*. Cleaved PARP and cleaved caspase 3 levels (ImageJ signal intensities) were first normalized to the corresponding pre-PARP and pre-caspase 3 levels, respectively, and then normalized to the si-control condition without Dox (−) and H_2_O_2_ treatment (0 μM). *E*, apoptosis in HEK293 cells expressing iNOS, eNOS, or nNOS was measured using the RealTime-Glo Annexin V Apoptosis Assay after H_2_O_2_ treatment. *F*, cleavage of PARP and caspase 3 in HEK293 cells expressing iNOS, eNOS, or nNOS after H_2_O_2_ treatment. Expression levels of iNOS, eNOS, and nNOS are shown in the *lower panel*. β-tubulin and GAPDH were used as a loading control. In *B* and *F*, spliced blots from the same gel for pre-caspase/cleaved caspase 3 or eNOS/iNOS/nNOS are indicated. The spliced blots were merged during enhanced chemiluminescence exposure for comparison. *G* and *H*, quantification of cleaved PARP and cleaved caspase 3 level in *F*. The cleaved PARP level and cleaved caspase 3 levels of all samples (ImageJ signal intensity) were first normalized to the corresponding pre-PARP and pre-caspase 3 levels, respectively, and then normalized to the levels of HEK293 cells without NOS expression (−) and H_2_O_2_ treatment (0 μM). Results in *A*, *C*, *D*, *G*, and *H* are presented as mean ± SD. Each plot represents one biological repeat. One-way ANOVA with Tukey's *post hoc* test was used to detect the significance. ∗*p* < 0.05, ∗∗*p* < 0.01, and ∗∗∗∗*p* < 0.0001. eNOS, endothelial nitric oxide synthase; H_2_O_2_, hydrogen peroxide; HEK, human embryonic kidney cell line; iNOS, inducible nitric oxide synthase; nNOS, neuronal nitric oxide synthase; PARP, poly(ADP-ribose) polymerase; STRAP, serine–threonine kinase receptor–associated protein.

The STRAP–ASK1–MKK–p38 pathway plays a critical role in modulating H_2_O_2_-induced apoptosis. To investigate the role of STRAP S-nitrosylation in this process, we examined how STRAP affects apoptosis under low concentrations of H_2_O_2_ (30 μM and 100 μM), which can induce significant apoptosis (22). Overexpression of iNOS markedly increased apoptosis by 2.9-fold at 30 μM H_2_O_2_ and 3.4-fold at 100 μM H_2_O_2_ compared with cells without iNOS expression (Fig. 4*A*), supporting a role for iNOS-derived NO in promoting apoptosis. To assess the involvement of STRAP in iNOS-mediated apoptosis, we knocked down STRAP expression (Fig. 4*B*). STRAP knockdown significantly enhanced apoptosis compared with si-control cells (Fig. 4*A*), confirming its inhibitory role. Notably, in si-STRAP cells, iNOS overexpression led to only a modest increase in apoptosis—1.3-fold at 30 μM H_2_O_2_ and 1.4-fold at 100 μM H_2_O_2_—compared with si-STRAP cells without iNOS expression (Fig. 4*A*). These results indicate that STRAP is required for the full proapoptotic effect of iNOS.

In addition to quantifying apoptotic cell death, we examined the cleavage of two apoptotic markers: poly(ADP-ribose) polymerase (PARP) and caspase 3. Treatment with H_2_O_2_ induced cleavage of both PARP and caspase 3 in control HEK293 cells (Fig. 4, *B*–*D*). Similarly, either iNOS overexpression or STRAP knockdown increased cleavage of these proteins (Fig. 4, *B*–*D*), indicating that both iNOS and STRAP are crucial regulators of apoptosis. However, iNOS overexpression in si-STRAP cells did not further enhance PARP or caspase 3 cleavage (Fig. 4, *B*–*D*), again confirming that STRAP is required for iNOS-induced apoptosis.

Further analysis revealed that only iNOS—but not eNOS or nNOS—promoted apoptosis and induced cleavage of both PARP and caspase 3 (Fig. 4, *E*–*H*), suggesting that NO derived specifically from iNOS is the key regulator of STRAP activity in cell survival.

**Figure 5:**
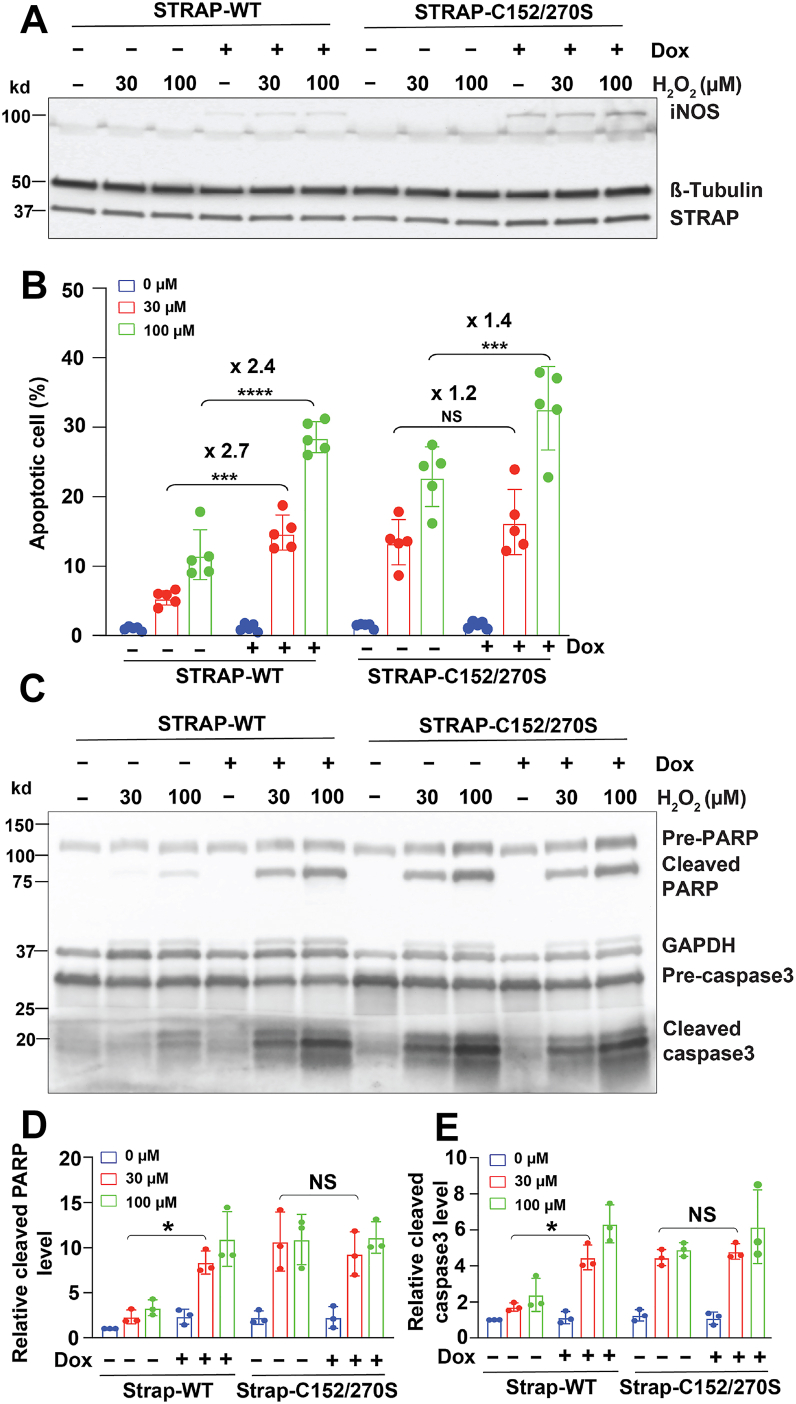
**Mutation of S-nitrosylation sites in STRAP alters H_2_O_2_-induced apoptosis.***A*, expression of iNOS and STRAP in HEK-STRAP-WT and HEK-STRAP-C152/270S cells. *B*, apoptosis in HEK-STRAP-WT and HEK-STRAP-C152/270S cells was measured using the RealTime-Glo Annexin V Apoptosis Assay. Cells were induced with doxycycline (Dox) (“+”) and treated with H_2_O_2_. All samples were normalized to the average apoptotic cell percentage of STRAP-WT without Dox induction (−) and H_2_O_2_ treatment (0 μM). *C*, cleavage of PARP and caspase 3 in HEK-STRAP-WT and HEK-STRAP-C152/270S cells following Dox induction (“+”) and H_2_O_2_ treatment. n = 3. *D*, quantification of cleaved PARP and cleaved caspase 3 levels in *C*. The cleaved PARP level and cleaved caspase 3 level of all samples (ImageJ signal intensity) were first normalized to the corresponding pre-PARP and pre-caspase 3 levels, respectively, and then normalized to the levels of STRAP-WT without Dox induction (−) and H_2_O_2_ treatment (0 μM). Results in *B*, *D*, and *E* are presented as mean ± SD. Each plot represents one biological repeat. One-way ANOVA with Tukey's *post hoc* test was used to detect significance. ∗*p* < 0.05, and ∗∗∗*p* < 0.001 and ∗∗∗∗*p* < 0.0001. H_2_O_2_, hydrogen peroxide; HEK, human embryonic kidney cell line; iNOS, inducible nitric oxide synthase; PARP, poly(ADP-ribose) polymerase; STRAP, serine–threonine kinase receptor–associated protein.

To determine whether S-nitrosylation of STRAP modulates apoptosis, we examined H_2_O_2_-induced apoptosis in cells expressing STRAP-WT or SNO-deficient mutant of STRAP (STRAP-C152/270S) (Fig. 5*A*). In STRAP-WT cells, iNOS expression increased apoptosis by 2.7-fold at 30 μM H_2_O_2_ and 2.4-fold at 100 μM H_2_O_2_ compared with cells without iNOS expression (Fig. 5*B*), similar to previous results (Fig. 4*A*). In STRAP-C152/270S mutant cells, apoptosis was already elevated because of the mutant’s inability to interact with ASK1 and inhibit its activity (Fig. 5*B*). Importantly, iNOS overexpression in STRAP-C152/270S cells did not further increase apoptosis—1.2-fold at 30 μM H_2_O_2_ and 1.4-fold at 100 μM H_2_O_2_—compared with STRAP-C152/270S cells without iNOS expression (Fig. 5*B*), indicating that STRAP S-nitrosylation is a critical regulatory step. Consistent with these findings, iNOS overexpression increased PARP and caspase 3 cleavage in si-control cells but not in STRAP-C152/270S cells (Fig. 5, *C*–*E*), demonstrating that iNOS loses its partial ability to promote apoptosis in the absence of functional SNO sites on STRAP.

## Discussion

**Figure 6:**
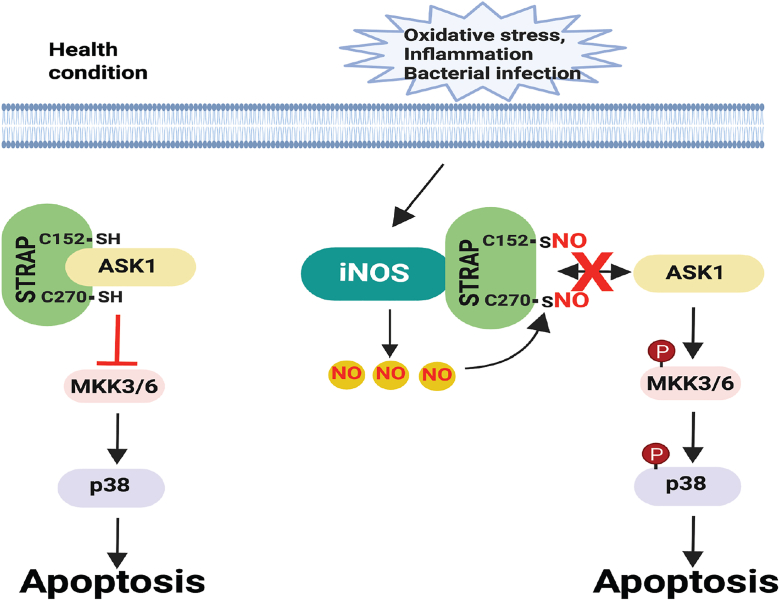
**Working model of S-nitrosylation STRAP in apoptosis.** Under healthy conditions, unmodified STRAP interacts with ASK1 and inhibits ASK1 activity, thereby deactivating the ASK1–MKK–p38 signaling pathway and suppressing apoptosis. However, under pathological stress conditions, such as oxidative stress, inflammation, or bacterial infection, iNOS is induced in response to these stimuli. NO produced by iNOS mediates the S-nitrosylation of STRAP. S-nitrosylated STRAP loses its ability to bind ASK1, resulting in the release and activation of ASK1, which in turn activates the MKK–p38 pathway and promotes apoptosis. ASK1, apoptosis signal–regulating kinase 1; iNOS, inducible nitric oxide synthase; MKK, mitogen-activated protein kinase kinase; NO, nitric oxide.

This is the first study to demonstrate that STRAP can be S-nitrosylated and that SNO-STRAP plays a critical role in regulating cellular sensitivity to apoptosis. Under normal healthy condition, STRAP interacts with ASK1 and inhibits its activity, thereby deactivating the ASK1–MKK–p38 signaling pathway and suppressing apoptosis. However, under pathological stress conditions, such as oxidative stress, inflammation, or bacterial infection, iNOS is induced in response to these stimuli. NO produced by iNOS mediates STRAP S-nitrosylation at Cys152 and Cys270. S-nitrosylated STRAP (SNO-STRAP) loses its ability to bind ASK1, resulting in the release and activation of ASK1, which in turn activates the MKK–p38 pathway and promotes apoptosis (Fig. 6). The iNOS–SNO-STRAP–ASK1 axis thus represents a novel mechanism by which iNOS regulates apoptosis.

In addition to interacting with ASK1, STRAP also binds to various partner proteins, including Nm23-H1, SMAD3, TGF-β, p53, and PDK1, and regulates a range of cellular processes, such as cell cycle progression, proliferation, differentiation, survival, and apoptosis (23, 24, 25, 26). In many cases, Cys152 and Cys270 are essential for these protein–protein interactions, such as MPK38 and Nm23-H1 (16, 24, 26). Beyond its role in apoptosis, iNOS is also involved in regulating the cell cycle, proliferation, and differentiation (27, 28). Therefore, it is plausible that STRAP S-nitrosylation mediated by iNOS modulates its interactions with other partner proteins, thereby influencing multiple cellular processes. Investigating the broader roles of SNO-STRAP and elucidating how its specific functions are mediated through changes in its interacting partners will be an important area for future research.

Although eNOS and nNOS can also generate NO, iNOS produces NO at much higher levels than eNOS and nNOS (6). In addition to the high levels of NO derived from iNOS, the interaction between iNOS and STRAP may directly facilitate S-nitrosylation of STRAP, indicating the important role of STRAP in the iNOS signaling pathway. iNOS expression is highly inducible in inflammatory, infectious, and metabolic disease states (29), suggesting that SNO-STRAP may serve as a context-dependent regulatory node in these diseases. In chronic inflammatory environments—such as atherosclerosis, diabetes, and certain autoimmune conditions—prolonged iNOS induction could promote sustained SNO-STRAP formation, leading to inappropriate suppression of ASK1 and reduced clearance of damaged cells. This may contribute to pathological cell survival and tissue dysfunction. Conversely, in conditions where excessive apoptosis is detrimental, such as neurodegeneration or ischemia–reperfusion injury, enhancement of SNO-STRAP formation might protect vulnerable cells by restraining ASK1 overactivation.

Our study also raises intriguing therapeutic possibilities. Targeting SNO level of STRAP—either by manipulating local NO production or by developing molecules that mimic or block the SNO modification—could provide a means to modulate ASK1 activity. Given that ASK1 is implicated in diverse diseases, including cardiovascular disorders, metabolic dysfunction, neurodegeneration, and cancer (30), the SNO-STRAP–ASK1 regulatory axis may represent a valuable and previously unrecognized therapeutic entry point. Future studies will be needed to evaluate how SNO dynamics vary across disease states and test whether pharmacological modulation of SNO-STRAP can produce meaningful benefits in relevant *in vivo* models.

Together, these findings expand our understanding of NO-mediated redox signaling and STRAP S-nitrosylation as a critical redox-sensitive modulator of ASK1-dependent apoptosis. By linking inflammatory NO production with the regulation of cellular stress responses, this work provides foundational insight into how apoptotic thresholds are established and suggests new strategies for restoring homeostasis in diseases characterized by dysregulated cell death.

## Experimental procedures

### Plasmids, cloning, and mutagenesis

The pcDNA-FLAG-STRAP and pcDNA-myc-ASK1 plasmids were obtained from GenScript. The pcDNA-FLAG-STRAP-C152S, C270S, C305S, and C340S mutants were generated using the QuikChange II Site-Directed Mutagenesis Kit (Agilent). The pTRE-iNOS construct was generated by inserting the iNOS complementary DNA into the pTRE vector (Takara) between the BglII and NdeI restriction sites. The iNOS complementary DNA was amplified by PCR using the primers 5′-CCTAGATCTATGGCCTGTCCTTGGAAATTTCTGT-3′ and 5′-CGTCATATGTTAGAGCGCTGACATCTCCAGGCTG-3′, with BglII and NdeI sites introduced at the ends. All plasmids were verified by DNA sequencing.

### Cell line generation, siRNA knockdown, and treatment

Tet-On–induced iNOS expression cell lines were generated using the Takara Tet-On 3G Inducible Expression System. Briefly, a stable Tet-On Advanced HEK293 cell line was first established. HEK293 cells were transfected with the Tet-On Advanced vector using PolyJet transfection reagent (SignaGen). After 2 weeks of G418 selection (500 µg/ml), resistant colonies were picked and transiently transfected with a pTRE-luciferase reporter. The colony showing the highest fold induction of luciferase expression upon Dox (1.0 μg/ml) treatment was selected for generating the iNOS-inducible line. The pTRE-iNOS construct was transfected into this Tet-On Advanced cell line with a linear hygromycin resistance marker (at a vector-to-marker molar ratio of 20:1), followed by 2 weeks of hygromycin selection (200 µg/ml). Hygromycin-resistant colonies were screened for Dox (1.0 μg/ml)-inducible iNOS expression, and the clone showing robust iNOS induction was used for phosphorylation and apoptosis assays.

To knock down endogenous STRAP in HEK293-iNOS cells, two custom siRNAs targeting the 3′ UTR of human STRAP (5′-GGGAAUACAUGAUAAAGUAUU-3′ and 5′-AAACAAGCAAGCAGAGAAAUU-3′) were purchased from Dharmacon. Reverse transfection was performed using Lipofectamine RNAiMAX (Invitrogen) in a master mix, which was then evenly distributed into wells to ensure equal cell numbers per well. For Annexin V-based apoptosis assays in 96-well plates, 5 pmol of STRAP siRNA was transfected into 2 × 10^4^ cells per well. For cleavage assays and phosphorylation assays in 6-well plates, 30 pmol of STRAP siRNA was transfected into 1 × 10^6^ cells per well. At 24 h post-transfection, Dox (1.0 μg/ml) and l-arginine (50 μM) were added to the medium to induce iNOS expression and NO production. After 24 h of induction, cells were subjected to H_2_O_2_ treatment and used for phosphorylation and apoptosis assays.

To replace endogenous STRAP in HEK293-iNOS cells with either STRAP-WT or STRAP-C152/270A, endogenous STRAP was knocked down using the same siRNAs, and expression plasmids encoding STRAP-WT or STRAP-C152/270A were cotransfected into cells. Reverse transfection was performed using Lipofectamine 2000 (Invitrogen) in a master mix and evenly distributed into wells to ensure equal cell numbers. For 96-well apoptosis assays, 5 pmol of STRAP siRNA and 0.2 μg of STRAP-WT or STRAP-C152/270A plasmid were transfected into 2 × 10^4^ cells per well. For 6-well cleavage and phosphorylation assays, 100 pmol of siRNA and 4 μg of plasmid were transfected into 1 × 10^6^ cells per well. After 24 h, Dox (1.0 μg/ml) and l-arginine (50 μM) were added to induce iNOS expression. After another 24 h, cells were subjected to H_2_O_2_ treatment and used for phosphorylation and apoptosis assays.

### Western blot analysis

Proteins were extracted from cells by sonication in RIPA buffer (Fisher) supplemented with 1 mM PMSF and a protease inhibitor cocktail. Lysates were clarified by centrifugation at 14,000*g* for 20 min at 4 °C, and protein concentrations were determined using the bicinchoninic acid (BCA) assay. Equal amounts of protein were resolved by SDS-PAGE using 4% to 20% Criterion Precast Midi Protein Gels and transferred to polyvinylidene difluoride (PVDF) membranes. Membranes were incubated overnight at 4 °C with primary antibodies, washed with PBS containing 0.1% Tween-20, incubated with horseradish peroxidase–conjugated secondary antibodies for 1 h at room temperature, washed again, and visualized using a KwikQuant digital imaging system. Protein band intensities were quantified by semiquantitative analysis using ImageJ software (National Institutes of Health).

For phosphorylation assays, a phosphatase inhibitor cocktail was added to the RIPA buffer. Phosphorylation levels were analyzed by SDS-PAGE, followed by immunoblotting using antibodies from Cell Signaling Technology, including phospho-MKK3 (9231S; 1:1000 dilution) and phospho-p38 (9215S; 1:1000 dilution), as well as a mouse anti-p97 antibody (10R-P104a; 1:2000 dilution) from Fitzgerald Industries. The PVDF membrane was stripped using Restore PLUS Western Blot Stripping Buffer (Thermo Scientific) for 10 min and reblocked with antibodies rabbit anti-MKK3 (8535S; 1:1000 dilution) and anti-p38 (8690S; 1:1000 dilution) from Cell Signaling Technology.

### Immunoprecipitation

To investigate the interaction between NOS isoforms and STRAP, plasmids pcDNA-eNOS, pcDNA-nNOS, or pcDNA-iNOS were coexpressed with pcDNA-FLAG-STRAP in HEK293 cells. To assess the interaction between STRAP and ASK1, plasmids pcDNA-FLAG-STRAP were coexpressed with pcDNA-myc-ASK1 in Dox-inducible HEK293-iNOS cells. iNOS expression was induced by adding Dox to the culture medium for 24 h. IP was performed using anti-FLAG M2 Affinity Gel (Sigma), whereas Protein G Sepharose (GE) coupled with mouse immunoglobulin G served as a negative control. Cells were lysed in EBC buffer (120 mM NaCl, 20 mM Tris–Cl, pH 8.0, 0.5 mM EDTA, 0.5% NP-40, 1 mM PMSF, and protease inhibitor cocktail). Lysates were clarified by centrifugation at 14,000*g* for 20 min at 4 °C (repeated twice). Two milliliters of the supernatant (2 mg/ml total protein) were precleared by incubation with 50 μl Protein G Sepharose (1:1 slurry) for 1 h at 4 °C. After centrifugation at 1000*g* for 1 min, the supernatant was transferred to fresh tubes and incubated with either 50 μl of Protein G Sepharose coupled with mouse immunoglobulin G or 50 μl of anti-FLAG agarose (1:1 slurry) for 5 h at 4 °C.

Beads were washed with NETN buffer, and bound proteins were eluted with 50 μl of 1× SDS loading buffer containing 5% 2-mercaptoethanol. Eluted proteins were separated by SDS-PAGE on 4% to 20% Criterion Precast Midi Protein Gels (Bio-Rad), transferred to PVDF membranes, and probed with the following antibodies: rabbit anti-eNOS (Cell Signaling, 32027S), rabbit anti-nNOS (Cell Signaling, 4231S), rabbit anti-iNOS (Santa Cruz, sc-8310), and rabbit anti-myc (Cell Signaling, 2278S).

### STRAP protein purification

STRAP-WT and STRAP-C152/270S proteins were purified from HEK293 cells. Briefly, cells were transfected with either pcDNA-FLAG-STRAP-WT or pcDNA-FLAG-STRAP-C152/270S plasmid using PolyJet transfection reagent (SignaGen) according to the manufacturer’s instructions and cultured for 48 h. Cells were then harvested in IP lysis buffer (as described above) supplemented with 0.5% Triton X-100 and a protease inhibitor cocktail. Cell lysis was performed by sonication, and the lysates were clarified by centrifugation at 14,000*g* for 20 min at 4 °C (repeated twice).

Anti-FLAG M2 Affinity Gel (Sigma) was equilibrated in IP lysis buffer, and the clarified lysates were applied to the Affinity Gel. After 4 h of incubation at 4 °C, the beads were washed five times with IP wash buffer. Bound proteins were eluted in 300 μl of kinase buffer (40 mM Tris, pH 7.5; 20 mM MgCl_2_; and 0.1 mg/ml bovine serum albumin) containing 100 μg/ml 3× FLAG peptide. Protein concentrations were determined using the BCA assay.

### *In vitro* ASK1 activity assay

*In vitro* ASK1 activity assays were performed using the ASK1 Kinase Enzyme System coupled with the ADP-Glo Kinase Assay (Promega). Briefly, 10 μl (10 ng/μl) of purified STRAP-WT or STRAP-C152/270S complex was incubated with either control Tris buffer or 500 μM CysNO (5 μl) for 30 min at 37 °C. Subsequently, a 10 μl reaction mixture containing ASK1 (15 ng), ATP (25 μM), and the substrate MBP (1 μg) was added. After a 60-min incubation at 25 °C, 25 μl of ADP-Glo Reagent was added. Following a 40-min incubation at 25 °C, 50 μl of kinase detection reagent was added, and luminescence was measured after an additional 30-min incubation at 25 °C using a Promega GloMax Luminometer.

### SNO-RAC

SNO-RAC was carried out as described previously (31). The cells were lysed in lysis buffer (1 mg/5 μl lysis buffer) containing 100 mM Hepes, 1 mM EDTA, 100 μM neocuproine (Hepes, EDTA, neocuproine [HEN]), 50 mM NaCl, 0.1% (v/v) Nonidet P-40, 0.2% S-methylmethanethiosulfonate as a free thiol-blocking agent, 1 mM PMSF, and protease inhibitors. After centrifugation (20,000*g*, 4 °C, 10 min, 2×), SDS and S-methylmethanethiosulfonate were added to the supernatants to 2.5% and 0.2%, respectively, and incubated at 50 °C for 20 min. Proteins were precipitated with −20 °C acetone and redissolved in 1 ml of HEN and 1% SDS. Precipitation of proteins was repeated with −20 °C acetone, and the final pellets were resuspended in HEN, 1% SDS buffer, and protein concentrations were determined using the BCA method. Total lysates (2 mg) were incubated with freshly prepared 50 mM ascorbate and 50 μl thiopropyl-Sepharose (50% slurry) and rotated end over end in the dark for 4 h. The bound SNO proteins were sequentially washed with HEN, 1% SDS, and then 10% HEN, 0.1% SDS buffers; SNO proteins were eluted with 10% HEN, 1% SDS, 10% β-meracaptoethanol, and analyzed by SDS-PAGE and immunoblotting with mouse STRAP antibody (Proteintech, 66712) or mouse M2 FLAG antibody (Sigma).

### Cell apoptosis assay

Cell apoptosis was measured using the RealTime-Glo Annexin V Apoptosis Assay (Promega). Phosphatidylserine, which becomes exposed on the outer leaflet of the cell membrane during apoptosis, was detected through annexin V binding, generating a luminescent signal. After cells were treated with H_2_O_2_ (30 μM or 100 μM) for 3 h in 96-well plates, 100 μl of 2× Detection Reagent was added to each well. The plate was shaken for approximately 30 s at 500 to 700 rpm to ensure proper mixing, and luminescence was measured after 10 min.

To assess the cleavage of apoptotic markers, PARP and caspase 3, cells were treated with H_2_O_2_ (30 μM or 100 μM) for 3 h in 6-well plates. Following treatment, cells were collected, lysed, and subjected to SDS-PAGE, followed by immunoblotting using Cell Signaling Technology antibodies: rabbit anti-PARP (9542S; 1:2000 dilution), anti–caspase-3 (9665S; 1:2000 dilution), anti–cleaved caspase-3 (9664S; 1:1000 dilution), and anti–GAPDH (2118S; 1:5000 dilution).

## Data availability

All data generated or analyzed during this study are included in this published article.

## Human and animal rights and informed consent

No animal or human subjects were used by the authors in this study.

## Conflict of interest

The authors declare that they have no conflicts of interest with the contents of this article.
